# Survival, health care resource utilization and expenditures of first-line treatments for multiple myeloma patients ineligible for transplant in Taiwan

**DOI:** 10.1371/journal.pone.0252124

**Published:** 2021-05-26

**Authors:** Chih-Ning Cheng, Shang-Yi Huang, Pei-Wen Lien, Shih-Ting Huang, Fang-Ju Lin

**Affiliations:** 1 Graduate Institute of Clinical Pharmacy, College of Medicine, National Taiwan University, Taipei, Taiwan; 2 Department of Internal Medicine, National Taiwan University Hospital, Taipei, Taiwan; 3 Takeda Pharmaceuticals Taiwan, Ltd, Taipei, Taiwan; 4 School of Pharmacy, College of Medicine, National Taiwan University, Taipei, Taiwan; 5 Department of Pharmacy, National Taiwan University Hospital, Taipei, Taiwan; University of Oxford, UNITED KINGDOM

## Abstract

**Background:**

We aimed to provide real-world information on survival, health care resource utilization (HCRU), and expenditures related to various first lines of therapy (1LOTs) in newly diagnosed multiple myeloma (NDMM) patients who were transplant ineligible (TI).

**Patients and methods:**

From the Taiwan National Health Insurance Database (2008–2016), we identified 1,511 NDMM-TI patients who had received 1LOT since June 2012. We categorized 1LOT regimens into four groups: bortezomib (V)+thalidomide (T), V, T, and non-V/T. Patients’ characteristics were collected. The overall survival (OS), event-free survival (EFS), frequencies of HCRU (hospitalization, visiting outpatient and emergency departments), and related expenditures within one year after commencement of the 1LOT were evaluated and compared.

**Results:**

The mean age of the included patients was 71.3 (SD 10.7) years, and 40.4% of patients had a CCI score ≥3. Most patients (747; 49.4%) were in the V+T group and, after adjusting for covariates, had a significantly longer OS (median, 22.2 months) and EFS (9.1 months) than those in the T group (12.6 and 4.5 months, respectively) and the non-V/T group (12.2 and 3.2 months, respectively), but they were mostly comparable with patients in the V group (23.8 and 6.6 months, respectively). Compared to those in the V+T group, patients in the T and non-V/T groups had 29% and 39% fewer outpatient visits and 15% and 24% lower total expenditure, respectively.

**Conclusion:**

Our real-world data consolidate evidence for the effectiveness of bortezomib-containing regimens as the 1LOT in NDMM-TI patients at the expense of more outpatient visits and higher total costs.

## Introduction

Multiple myeloma (MM) is characterized by proliferation of abnormal plasma cells with resultant organ damage (e.g., anemia, renal failure, and osteolytic bone lesions) and accounts for 10–15% of hematologic malignancies [1,2]. The incidence of MM has continuously increased across the world [3] including in Taiwan where the age-adjusted incidence of MM increased from 1.41 per 100,000 population in 2007 to 1.65 per 100,000 population in 2015 [3,4].

Although autologous hematopoietic stem cell transplantation (AuHSCT) and the introduction of several novel agents (e.g., bortezomib [V], thalidomide [T], and lenalidomide [R]) for MM treatment have been shown to improve the outcomes of MM patients, MM is still an incurable disease [5]. Notably, the majority of these treatment data come from clinical trials and thus are unlikely to reflect the real-world population, resulting in a gap between clinical trial efficacy and real-world effectiveness [6,7]. Furthermore, such improved outcomes were limited to mostly relatively young and fit MM patients since older and frail patients had lower utilization of AuHSCT and were less likely to be recruited into trials of novel drugs. For instance, a long-term follow-up (1994–2013) of MM survival in Switzerland showed substantial improvement in patients aged less than 75 years but only minimal changes in older patients [8]. A similar observation was noted in a US claims-based analysis: survival of MM patients was still inversely correlated with age in the novel agent era [9]. Herein, nearly two-thirds of MM patients were older than 65 years at diagnosis [2], and only approximately 15% of our MM patients had received AuHSCT according to recent surveys [4,10]. Moreover, we still observed a higher mortality in our AuHSCT-ineligible MM patients even in the novel agent era [4].

Therefore, we conducted this study to describe the characteristics of NDMM-transplant-ineligible (TI) patients and to investigate the impact of the first line of therapy (1LOT) on their clinical outcomes including event-free survival (EFS) and overall survival (OS). In addition, it is imperative to inspect the impact of these treatment options on health care resource utilization (HCRU) (e.g., clinic visits and hospital admissions) and expenditures.

## Methods

### Data source

This retrospective MM cohort study was conducted using the 2008–2016 subset of the National Health Insurance Research Database (NHIRD) in Taiwan. Taiwan has a population close to 24 million. The single-payer National Health Insurance (NHI) system that has been in place since March 1995 provides mandatory health insurance and services for nearly the entire population [11]. The NHIRD is a nationwide claims database that contains complete longitudinal claims data regarding diagnosis, medication records, records of outpatient and emergency room visits, and hospital admissions and covers 99.9% of the entire population in Taiwan [12]. In addition, the NHIRD can be linked to the Taiwan Cancer Registry (TCR) and Catastrophic Illness Registry (CIR) to verify the diagnosis of MM. The TCR, which was established in 1979, contains detailed information about new cancer cases, including the diagnosis, stage, and treatment. Since 2001, patients who have catastrophic illnesses are routinely registered in the CIR. The application of catastrophic illness certification requires formal review and is only qualified for those who have a rare or severe illness (e.g., malignancies). The NHI Administration also verifies the accuracy of the information stored in the database through random reviews of one per 100 ambulatory cases and one per 20 inpatient claim cases as well as patient interviews. The National Death Registry in Taiwan was used to determine mortality for the included patients. In these databases, all patient information is deidentified, and the analysis results were carefully reviewed by the Health and Welfare Data Center to assure anonymity. The study protocol was approved by the Research Ethics Committee of National Taiwan University Hospital (REC No. 201710029W). The database was accessed since June 2018.

### Study population

Using the NHIRD from 2008–2016, we identified MM patients with at least two outpatient codes or one inpatient diagnostic code for MM (International Classification of Diseases, Ninth Revision, Clinical Modification [ICD-9-CM] codes 203.0x) between January 1, 2009, and December 31, 2015. The date of the first 203.0x coding was defined as the cohort entry date and referred as the date of MM diagnosis. For the cohort study, the end date of follow-up was December 31, 2016. Patients were further excluded if they (i) had an MM diagnosis within one year before the cohort entry date to ensure those who enrolled had newly diagnosed MM (NDMM); (ii) had never received MM treatment during the study period; (iii) had received allogeneic HSCT during the study period; (iv) had ever intended to or had received AuHSCT, determined by ever having mobilization, collection or cryopreservation of hematopoietic stem cells (HSCs); or (v) had the 1LOT before June 2012 while V was not yet reimbursed for the 1LOT in Taiwan.

### Treatment and treatment lines

In Taiwan, bortezomib (V) was the first proteasome inhibitor (PI) reimbursed for MM treatment in June 2007, initially as the second line of therapy (2LOT) and subsequently as the 1LOT since June 2012. However, bortezomib is maximally reimbursed for a total of 8 cycles for each insured patient (reimbursement cap) regardless of when or how the drug is used. The immunomodulatory drug (IMiD) thalidomide (T) was approved in August 2007 and then reimbursed as a 1LOT since July 2009. Lenalidomide (R), another IMiD, was approved and reimbursed for use in patients with treatment failure with the 1LOT since December 2012, with a reimbursement cap of 18 cycles, and subsequently as the 1LOT only in TI patients since February 2020, with a reimbursement cap of 24 cycles throughout different lines. To date, there are no restricted criteria for MM patients to receive autologous hematopoietic stem cell transplantation (AuHSCT); it can be applied at the discretion of the patients themselves and the treating physicians [13].

The anti-MM medications collected in this study and their abbreviations/Anatomical Therapeutic Chemical (ATC) codes are listed in S1 Table. The 1LOT regimens were further categorized into four treatment groups: (i) V+T-based; (ii) V-based (without T); (iii) T-based (without V); and (iv) non-V/T-based treatments. The details of various regimens among these four groups are shown in S2 Table. The date for 1LOT commencement (date of the first prescription) was defined as the index date. All anti-MM medications prescribed within 60 days (i.e., eligible period) after the index date were considered the same LOT [14]. Any new anti-MM medications prescribed after the eligible period and any same medication(s) prescribed within the prior regimen but restarted after more than 90 days since the last day of prior use (i.e., gap period) were considered the subsequent LOT.

### Survival, health care resources utilization, and costs

Patients were followed up from the index date until death, end of the study period (December 31, 2016), or disenrollment from the database, whichever occurred first. The length of OS was defined from the index date to the date of death due to any cause. The length of event-free survival (EFS) was defined from the index date to the date of commencement of the 2LOT or death due to any cause. Health care resource utilization (HCRU) was defined as the frequencies of visiting the outpatient department (OPD), emergency room (ER), and inpatient department (IPD; hospitalization) within one year since the index date. Patients who changed to a subsequent line of treatment or died within a year were censored. The total costs and costs related to OPD, ER, and IPD were presented per patient per month (PPPM). The IPD-associated costs were further separated into drug-related and non-drug-related costs. All costs are presented in US dollars ($) at the currency exchange rate of 1 USD ($) = 31.04 New Taiwan Dollars (NTDs).

### Covariates

Age and MM stage (Durie-Salmon staging [DSS]) at the cohort entry date, sex, accessibility to AuHSCT, and selected comorbidities (full list provided in S3 Table) were adjusted for in the outcome analysis, while the comorbidities of each patient were collected within one year prior to the cohort entry date. The Charlson Comorbidity Index (CCI; S4 Table) was also calculated and adjusted for in the analysis.

### Statistical analysis

For comparison, the Chi-square test and ANOVA were used for categorical and continuous variables, respectively. The OS and EFS were estimated by Cox proportional hazards models. In the time-to-event analysis, living patients were censored at either disenrollment from the health insurance program or the end of the study period, whichever came first. In all the outcome regression models, the V+T group was selected as the reference group. The HCRU of the four treatment groups was compared using negative binomial regression, and the rate ratios (RR) of the OPD, ER, and IPD visits compared with that of the reference group are presented. Costs were calculated by a generalized linear model with gamma distribution, and cost ratios (CRs) to the reference group were expressed and compared. To handle excess zeros in the costs of OPD, ER, and IPD visits, a two-stage model, including a logistic regression model and a generalized linear model with gamma distribution, was used with a 95% confidence interval (CI) estimated by bootstrapping. All the statistical analyses were performed with SAS 9.4 (SAS Institute Inc., Cary, NC, USA).

## Results

### Study population

**Fig 1:**
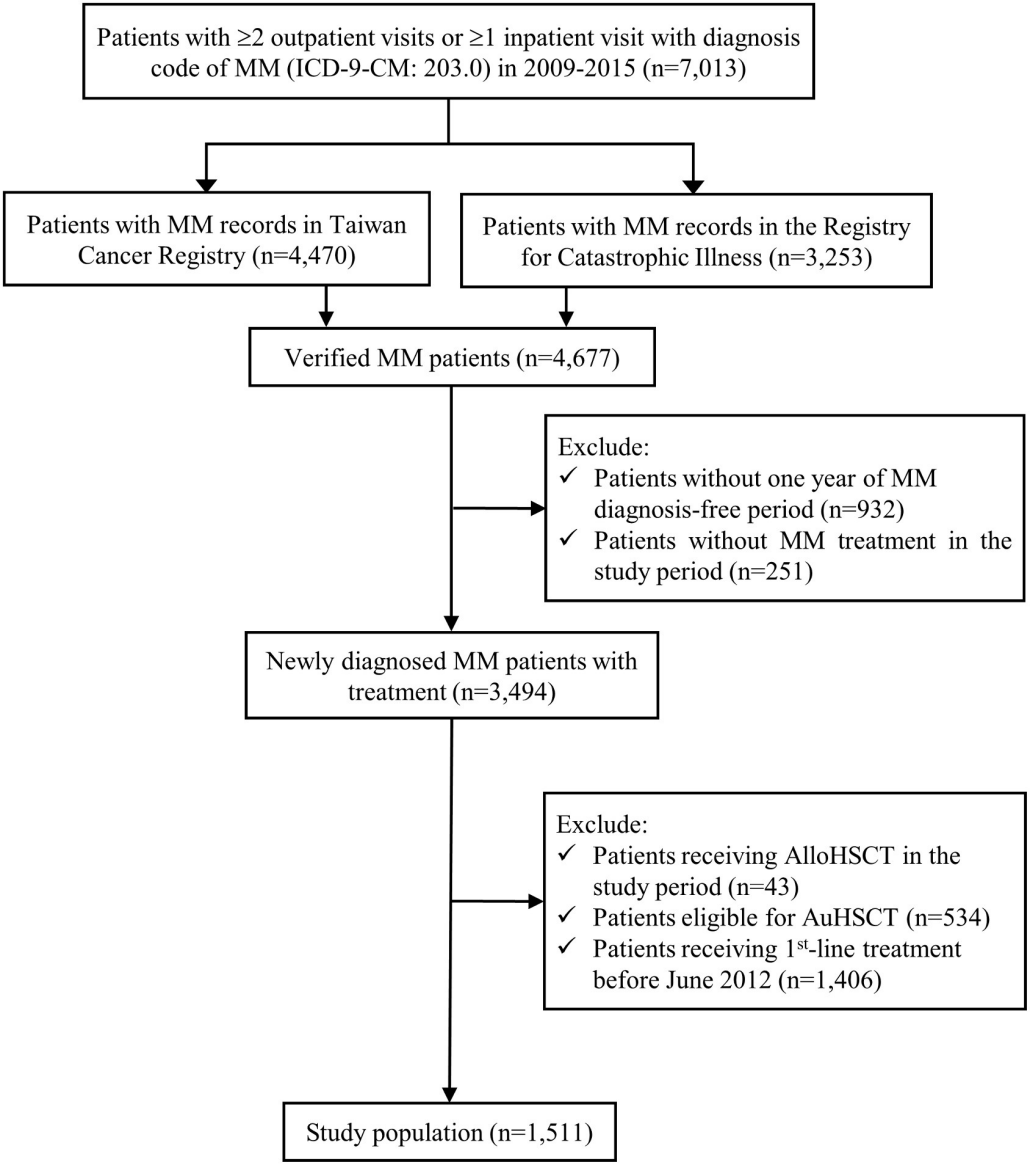
Flow chart of study population selection. AlloHSCT = allogeneic hematopoietic stem cell transplantation; AuHSCT = autologous hematopoietic stem cell transplantation; MM = multiple myeloma.

Through the patient selection process (Fig 1), we identified 1,511 NDMM-TI patients. These patients had commenced their 1LOT since June 2012 and had been followed until December 31, 2016.

### Patient characteristics and treatment groups

The baseline characteristics of the entire cohort are listed in Table 1. The patients had a mean age of 71.3 years (SD 10.7). At the cohort entry date, 40.4% of the patients had a CCI score of three or more (≥3). The most common comorbidities included cardiovascular disease (CVD; 32.0%), diabetes mellitus (DM; 25.7%), arthritis (24.3%), osteoporosis (17.9%), and chronic obstructive pulmonary disease (COPD; 11.1%). Among 898 (59.4%) patients with a known DSS, 668 (74.4%) patients had DSS III disease.

**Table 1:** Baseline patient characteristics.

Characteristics, n (%)	Total	V+T-based	V-based	T-based	Non-V/T-based	*p*-value*
**Number of patients**	1,511 (100.0)	747 (49.4)	303 (20.1)	321 (21.2)	140 (9.3)	
**Age (years) when receiving the 1LOT**						
Mean ± SD	71.3 ±10.7	69.5± 10.6	73.8± 9.4	72.7± 10.2	73.0± 12.3	< .001
<20	0 (0.0)	0 (0.0)	0 (0.0)	0 (0.0)	0 (0.0)	< .001
20–49	50 (3.3)	29 (3.9)	3 (1.0)	11 (3.4)	7 (5.0)	
50–64	348 (23.0)	215 (28.8)	47 (15.5)	59 (18.4)	27 (19.3)	
65–79	774 (51.2)	368 (49.3)	177 (58.4)	170 (53.0)	59 (42.1)	
≥80	339 (22.4)	135 (18.1)	76 (25.1)	81 (25.2)	47 (33.6)	
**Female**	680 (45.0)	334 (44.7)	141 (46.5)	140 (43.6)	65 (46.4)	0.878
**Durie-Salmon staging**						
Stage I	97 (6.4)	31 (4.2)	21 (6.9)	33 (10.3)	12 (8.6)	< .001
Stage II	133 (8.8)	67 (9.0)	20 (6.6)	35 (10.9)	11 (7.9)	
Stage III	668 (44.2)	356 (47.7)	155 (51.2)	122 (38.0)	35 (25.0)	
Missing	613 (40.6)	293 (39.2)	107 (35.3)	131 (40.8)	82 (58.6)	
**Accessible to AuHSCT**	1091 (72.2)	538 (72.0)	221 (72.9)	238 (74.1)	94 (67.1)	0.479
**Charlson Comorbidity Index**						
0	325 (21.5)	178 (23.8)	61 (20.1)	52 (16.2)	34 (24.3)	0.011
1	278 (18.4)	140 (18.7)	61 (20.1)	62 (19.3)	15 (10.7)	
2	297 (19.7)	142 (19.0)	56 (18.5)	78 (24.3)	21 (15.0)	
≥3	611 (40.4)	287 (38.4)	125 (41.3)	129 (40.2)	70 (50.0)	
**Comorbidities**						
Cardiovascular disease	484 (32.0)	209 (28.0)	117 (38.6)	105 (32.7)	53 (37.9)	0.003
Coronary heart disease	317 (21.0)	134 (17.9)	81 (26.7)	67 (20.9)	35 (25.0)	0.009
Congestive heart failure	229 (15.2)	99 (13.3)	59 (19.5)	50 (15.6)	21 (15.0)	0.088
Arrhythmia	152 (10.1)	70 (9.4)	38 (12.5)	29 (9.0)	15 (10.7)	0.408
Type 2 diabetes mellitus	389 (25.7)	183 (24.5)	83 (27.4)	84 (26.2)	39 (27.9)	0.707
Peripheral neuropathy	151 (10.0)	68 (9.1)	37 (12.2)	35 (10.9)	11 (7.9)	0.345
Ischemic stroke	61 (4.0)	30 (4.0)	10 (3.3)	13 (4.1)	8 (5.7)	0.696
Venous thromboembolism	14 (0.9)	6 (0.8)	NP^†^	NP^†^	4 (2.9)	0.157
Osteoporosis	270 (17.9)	131 (17.5)	55 (18.2)	61 (19.0)	23 (16.4)	0.907
Chronic obstructive pulmonary disease	168 (11.1)	72 (9.6)	34 (11.2)	39 (12.2)	23 (16.4)	0.112
Arthritis	367 (24.3)	183 (24.5)	73 (24.1)	79 (24.6)	32 (22.9)	0.978
Rheumatoid arthritis	24 (1.6)	9 (1.2)	5 (1.7)	NP^†^	NP^†^	0.488
Osteoarthritis	347 (23.0)	175 (23.4)	69 (22.8)	73 (22.7)	30 (21.4)	0.962

*P-values were calculated with the χ^2^ test for categorical variables and the t-test for continuous variables.

^†^To protect the anonymity of patients, data with a small sample size were not allowed to be provided (NP).

1LOT = first lines of therapy; AuHSCT = autologous hematopoietic stem cell transplantation.

The most common 1LOT group was V+T-based treatment (49.4%) followed by T-based (21.2%), V-based (20.1%), and non-V/T-based treatments (9.3%) (Table 1). The commonly used regimens in each treatment group are provided in S5 Table. Among the four treatment groups, patients in the V+T group were significantly younger and more likely to have DSS III disease. More patients in the V and non-V/T groups had comorbid CVD, especially coronary heart disease. More patients in the non-V/T group had a CCI score ≥3.

### Survival

**Fig 2:**
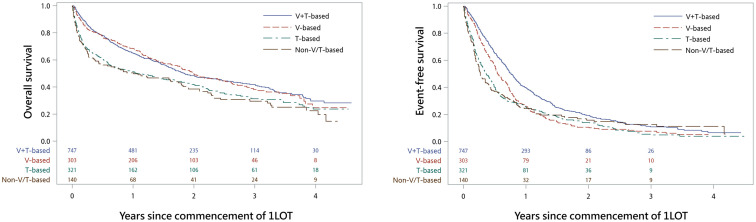
Overall survival (A) and event-free survival (B) of the 1LOT groups. In the analysis of event-free survival, the number of surviving patients at four years were less than three in some of the treatment groups; therefore, the data are not shown to protect the anonymity of the patients.

The median follow-up for the entire study cohort was 32.5 months (95% CI 30.9–33.8 months). The median OS was 21.1 months and was 22.2, 23.8, 12.6, and 12.2 months for patients in the V+T-based, V-based, T-based, and non-V/T-based treatment groups, respectively (Table 2; Fig 2). After adjusting for covariates, patients in the T group and non-V/T group still had significantly higher all-cause mortality than those in the reference group (T group: adjusted HR [aHR] 1.42 [95% CI 1.20–1.68; *p* < .001]; non-V/T group: aHR 1.55 [1.24–1.95; *p* < .001]) (Table 2). Old age (≥80 years), male gender, DSS II/III, CCI ≥3, and comorbid CVD were also associated with worse OS (S6 Table). The median EFS of the 1LOT for the entire cohort was 7.1 months. Patients in the V+T group had the longest EFS (median, 9.1 months) compared with the other treatment groups, and the difference remained significant after covariate adjustment (Table 2). Age ≤50 years, female gender, DSS I, and CCI = 0 were the factors also associated with a longer EFS (S7 Table).

**Table 2:** Comparison of overall survival and event-free survival among the 1LOT groups.

Event	Group	No. of events	No. of patients	Event rate (per 10 person-years)	Median time to event (months)	Crude		Adjusted	
						HR (95% CI)	*p*-value	HR (95% CI)	*p*-value
Death (Analysis of overall survival)	V+T-based	412	747	3.49	22.2	Reference		Reference	
	V-based	170	303	3.48	23.8	1.01 (0.85–1.21)	0.904	0.96 (0.80–1.16)	0.693
	T-based	216	321	4.69	12.6	**1.38 (1.17–1.63)**	**< .001**	**1.42 (1.20–1.68)**	**< .001**
	Non-V/T-based	98	140	5.28	12.2	**1.53 (1.23–1.91)**	**< .001**	**1.55 (1.24–1.95)**	**< .001**
Death or commencement of second line therapy (Analysis of event-free survival)	V+T-based	613	747	8.30	9.1	Reference		Reference	
	V-based	273	303	11.57	6.6	**1.35 (1.17–1.56)**	**< .001**	**1.32 (1.14–1.53)**	**< .001**
	T-based	293	321	12.64	4.5	**1.56 (1.35–1.79)**	**< .001**	**1.63 (1.41–1.88)**	**< .001**
	Non-V/T-based	120	140	11.56	3.2	**1.49 (1.23–1.82)**	**< .001**	**1.62 (1.32–1.98)**	**< .001**

CI = confidence interval; HR = hazard ratio.

### Health care resource utilization

Within one year following the index date, the median number of OPD visits was 19 (interquartile range [IQR], 6–33) for the entire study cohort. The related HCRU among each treatment group is shown in Table 3 as the RR and adjusted RR (aRR) compared with the reference group. Patients in the T group and non-V/T group had significantly fewer OPD visits with aRRs of 0.71 and 0.61, respectively (both *p* < .001). On the other hand, patients in T group and non-V/T group had comparable IPD visits (aRR, 1.16 and 1.22, respectively). Patients in the V group and non-V/T group had significantly fewer ER visits (aRR, 0.80 and 0.66, respectively; both *p* < .05).

**Table 3:** Crude and adjusted rate ratios of health care resource utilization among different 1LOT groups.

	Group	No. of visits, Mean ± SD	Rate per year	Crude		Adjusted	
				RR (95% CI)	*p*-value	RR (95% CI)	*p*-value
**Outpatient visits**	V+T-based	27.2 ± 19.3	38.06	Reference		Reference	
	V-based	22.4 ± 17.4	37.14	0.96 (0.89–1.04)	0.366	0.93 (0.86–1.01)	0.091
	T-based	14.2 ± 13.8	26.27	**0.75 (0.69–0.81)**	**< .001**	**0.71 (0.66–0.77)**	**< .001**
	Non-V/T-based	11.8 ± 14.6	21.72	**0.62 (0.55–0.70)**	**< .001**	**0.61 (0.54–0.68)**	**< .001**
**Inpatient visits (hospitalizations**)	V+T-based	2.3 ± 2.8	5.15	Reference		Reference	
	V-based	1.7 ± 2.1	4.96	0.86 (0.73–1.00)	0.056	0.86 (0.73–1.01)	0.060
	T-based	1.5 ± 1.4	10.90	1.15 (0.97–1.36)	0.103	1.16 (0.98–1.36)	0.081
	Non-V/T-based	1.6 ± 1.6	14.44	**1.28 (1.01–1.62)**	**0.040**	1.22 (0.97–1.54)	0.083
**Emergency room visits**	V+T-based	1.7 ± 2.5	3.14	Reference		Reference	
	V-based	1.3 ± 1.9	3.31	0.83 (0.68–1.02)	0.073	**0.80 (0.65–0.97)**	**0.027**
	T-based	1.0 ± 1.4	3.40	0.95 (0.77–1.18)	0.661	0.89 (0.73–1.10)	0.297
	Non-V/T-based	0.8 ± 1.4	3.04	0.73 (0.53–1.00)	0.051	**0.66 (0.48–0.90)**	**0.009**

CI = confidence interval; RR = rate ratio.

### Expenditures and cost ratios

**Fig 3:**
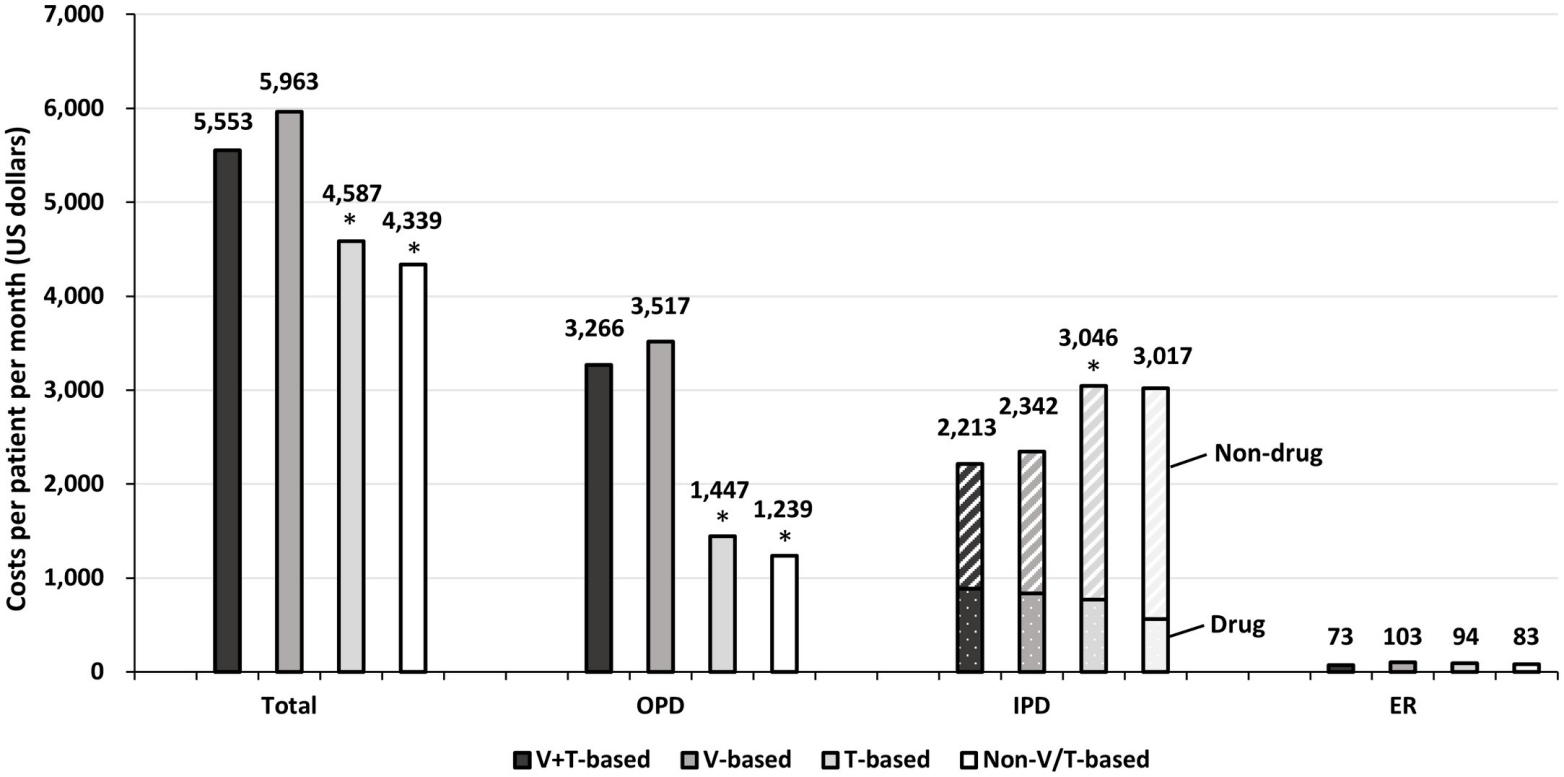
Average cost (per patient per month) within one year after commencement of the 1LOT. The asterisk (*) represents that the cost ratio (compared with the V+T-based group) achieved a significant difference. The bar with white slanted lines represents the IPD non-drug-related costs, and the bar with white dots represents the IPD drug-related costs. ER = emergency room; IPD = hospitalizations; OPD = outpatient.

The mean total expenditures (PPPM) for patients in the four groups, V+T, V, T and non-V/T, were $5,553, $5,963, $4,587, and $4,339, respectively (Fig 3). The total expenditures were significantly lower in the T and non-V/T groups compared to the V+T group (T group: adjusted cost ratio [aCR] 0.85 [95% CI, 0.77–0.95]; non-V/T group: aCR 0.76 [95% CI, 0.65–0.88]) (S8 Table). No significant difference was found in the expenditures for ER visits among these groups. IPD costs were significantly higher for patients in the T group (aCR 1.33 [95% CI, 1.05–1.68]) and were mainly contributed by non-drug-related costs. The drug-related IPD costs were also significantly lower in the T group (aCR, 0.73 [95% CI, 0.53–0.99]) and the non-V/T group (aCR, 0.55 [95% CI, 0.39–0.75]).

## Discussion

Based on the nationwide claims database (2008–2016), we identified 1,511 AuHSCT-ineligible NDMM patients who had commenced their 1LOT since June 2012; to date, this is a sizable cohort in a real-world setting and in the era of novel agents. Only a few similar claims-based studies have focused on the TI population [15,16]. The other studies, including some MM-specific registries, simply evaluated elderly MM patients but usually did not exclude those who were AuHSCT eligible (TE) or even never had a 1LOT [9,17–19]. In this study, to identify a true TI population, we included patients who had no administrative codes related to either transplant procedures or harvesting/cryopreservation of autologous hematopoietic stem cells (excluding not only patients who underwent AuHSCT but also those who ever intended to undergo AuHSCT). We also assessed the OS, HCRU, and costs related to the various 1LOTs. These assessments could be generally accurate since we linked patient data from the NHIRD with the death registry in Taiwan, which enabled us to collect comprehensive information regarding MM treatments and deaths wherever they occurred in Taiwan [12]. Furthermore, the MM patient selection criteria used in this study were comparable to those that have been validated recently [20].

To our knowledge, this is the first study that provides real-world data for NDMM-TI patients in the era of novel agents. The median OS was 21.1 months in our nationwide study cohort. An earlier study (2000–2012) also reported a similar OS of 22 months in NDMM-TI patients from a single medical center in Taiwan [21]. The OS here was much shorter than that in other claims-based studies that focused on elderly NDMM patients without fully excluding TE patients (ranging from 32.2 to 50.8 months) [9,17,19,22]. The more comorbidities and advanced disease status in our NDMM-TI patients might have led to this difference. In our study cohort, 73.7% of patients were older than 65 years, nearly 60% of patients had a CCI ≥2, and at least one-quarter to nearly one-third of patients had comorbidities such as CVD, DM, and arthritis. In contrast, two studies using the U.S. SEER database (2000–09, 2007–11) to evaluate elderly NDMM patients showed that 36% and 47.1% of patients had a CCI≥2. [9,18] In a German registry, as many as 63.5% of NDMM patients without AuHSCT in the 1LOT had a CCI = 0 [22]. We also observed a higher proportion of DSS III disease (74.4%) among our patients. In the EMMOS (multinational Europe, Middle East and Africa Multiple Myeloma Observational Study) and a study of a German registry, DSS III disease accounted for 65.4% and 59.6%, respectively, of NDMM patients who did not receive AuHSCT [22,23]. In addition, these findings highlight the need to enhance awareness and early diagnosis of MM in our Taiwanese population. Furthermore, unlike 15% of NDMM patients in Europe who might be enrolled in clinical trials as the 1LOT [19], we had only very few patients who would be eligible based on standard inclusion criteria.

Overall, the EFS observed in our study was shorter than the time to next treatment (TTNT) of various 1LOTs evaluated in registry studies [23]. However, because the exact date for disease progression or switching to the next LOT could not be clearly identified in this claims-based study, the results of these studies may not be directly comparable. On the other hand, because of the NHI reimbursement cap for novel agents (e.g., bortezomib and lenalidomide), patients might have to discontinue therapy before the maximum response is reached, which may partly explain the shorter EFS for the V+T group and V group in this study than those in other claims-based studies [15,16]. We found there was a tendency that novel agents, especially bortezomib, were reserved for later LOTs. Actually, among the 2LOT and 3LOT, there were still 34.9% and 21.0% V+T-based regimens, respectively, and 15.7% and 12.1% V-based regimens, respectively (unpublished data), suggesting that some physicians might preserve bortezomib for disease relapse/progression or even initiate bortezomib treatment after that.

Currently, a triplet combination consisting of a PI, an IMiD, and dexamethasone is the mainstream of the 1LOT for NDMM patients [5]. However, we observed a longer EFS for patients in the V+T group but only a comparable OS compared with those in the V group, which might challenge the need for V+T combination in NDMM-TI patients. In fact, V-containing regimens in the 1LOT, including the V+T and V groups, resulted in a longer EFS (median, 9.1 and 6.6 months, respectively) and OS (median, 22.2 and 23.8 months, respectively) than those in the other treatment groups without V-containing regimens (i.e., T and non-V/T groups), suggesting the important role of PI in the treatment of these patients regardless of a triplet or doublet combination. Other plausible explanations may also be considered. First, the subsequent treatment after the 1LOT can also contribute to OS [7]; however, this was not evaluated in our study. Second, TI patients are usually older and frail; thus, reduced-intensity regimens could have been more tolerable [24]. In the UPFRONT trial comparing VMP, VTD, and VD regimens for TI patients in community practice, VD showed a comparable OS with fewer adverse events than VMP and VTD [25], indicating that more treatment is not always better [24]. In addition, it is well known that many toxicities related to thalidomide may cause patients to reduce the dose or even discontinue the T-containing regimens [26]. An Australian study focusing on CyBorD followed by a sequential VTD approach showed that 23.5% of patients on VTD needed to withdraw treatment due to thalidomide-related toxicities [27]. In that regard, reimbursement scheme optimization that allows early use of more potent but less toxic agents should be considered to improve drug compliance in NDMM-TI patients. In a UK real-world study [28], the continuous use of lenalidomide in the 1LOT has been demonstrated to provide better outcomes than the use of major T-containing regimens (e.g., CTD and aCTD).

In this study, we also evaluated the HCRU and related expenditures in NDMM-TI patients using the nationwide claims database. Within one year after commencement of the 1LOT, patients using T-based and non-V/T-based regimens had 30–40% fewer OPD visits than patients using V-containing regimens (i.e., V+T and V groups). This may be because bortezomib is administered via intravenous or subcutaneous injection. In contrast, patients in the T groups had 33% higher hospitalization related costs than those in the V+T group. Although the reason was unclear, it might be due to poor disease controlor scheduled chemotherapy. It is well known that increased hospitalization related costs could aggravate the burden of disease and might negatively impact patients’ quality of life [29]. Furthermore, patients in the V group and non-V/T group had 20% and 34% fewer ER visits than patients in the V+T group, respectively. Bortezomib and thalidomide, alone or in combination (e.g., VTD, S5 Table), have been reported to be associated with a higher risk of cardiac adverse events, especially heart failure [30–32], as well as other common adverse effects including hematological toxicity, infections, and gastrointestinal complications [26]. Although not studied, one might hypothesize that the more ER visits observed in the V+T group could result from the greater number of adverse events, particularly from the coadministration of T because significantly fewer ER visits were observed in the V group. With respect to cost, we also found that the average total cost (including drug- and nondrug-related costs) was slightly lower in the V+T group than in the V group, while both were higher than those in the T and non-V/T groups. These observations again challenge the feasibility of the V+T combination as the 1LOT in NDMM-TI patients. More studies would be needed to confirm our findings.

This study has some limitations. First, similar to many other claims database studies, there is a lack of critical clinical information on disease severity and/or prognosis, such as the results of cytogenetic analysis, International Staging System (ISS), and revised ISS [5]. For estimating progression-free survival or TTNT, we could only define the LOT with specified periods (e.g., eligible period and gap period). Without laboratory and examination results, it is difficult to determine the treatment response or the exact date of disease progression. Second, we only included patients who commenced their 1LOT after bortezomib was reimbursed for the 1LOT, resulting in a relatively short follow-up in this study. Third, information on medications that were either paid for by the patients themselves, offered through special programs of a pharmaceutical company, or provided when patients were participating in clinical trials was missing from the database. Finally, our data indicated that nearly 30% of the patients were never treated in hospitals where AuHSCT is accessible; therefore, some patients in our cohort might still have been eligible for a transplant.

## Conclusion

In conclusion, our study showed prolonged survival associated with V-containing regimens as the 1LOT for NDMM-TI patients in a real-world setting. Although regimens containing both V and T might prolong the EFS, the OS was comparable to that of V-containing regimens without T. V-containing regimens also substantially increased the number of outpatient visits and related costs but partly compensated with lower inpatient expenditures as compared to T-based and non-V/T-based regimens. This study provides a rationale for conducting further research on the cost effectiveness of different novel agent combinations in real-world settings.

## Supporting information

S1 Table Anti-MM medications. (PDF)

S2 Table Medications within different regimens among treatment groups. (PDF)

S3 Table Selected comorbidities and associated International Classification of Disease, 9th and 10th editions, Clinical Modification (ICD-9-CM and ICD-10-CM) codes. (PDF)

S4 Table Charlson Comorbidity Index (CCI) and related diagnosis codes. (PDF)

S5 Table Common regimens in each first-line treatment group. (PDF)

S6 Table Full Cox proportional hazards model analysis for overall survival (OS). (PDF)

S7 Table Full Cox proportional hazards model analysis for event-free survival (EFS). (PDF)

S8 Table One-year actual cost, predicted cost, and cost ratio after commencement of first-line treatment. (PDF)
