# Supplementary material for: Survival, health care resource utilization and expenditures of first-line treatments for multiple myeloma patients ineligible for transplant in Taiwan
Source: PLoS One. 2021 May 26;16(5):e0252124. doi: 10.1371/journal.pone.0252124 (PMC8153459; doi:10.1371/journal.pone.0252124)
Supplement: S1 Table — (PDF) [file pone.0252124.s001.pdf]

Supplementary Table 1. Anti-MM medications.

Drugs	Abbreviation	ATC code
bortezomib	V	L01XX32
thalidomide	T	L04AX02
lenalidomide	R	L04AX04
cyclophosphamide	C	L01AA01
doxorubicin	A	L01DB01
melphalan	M	L01AA03
etoposide	E	L01CB01
cisplatin	p	L01XA01
vincristine	v	L01CA02
dexamethasone	D	H02AB02
