# Supplementary material for: Survival, health care resource utilization and expenditures of first-line treatments for multiple myeloma patients ineligible for transplant in Taiwan
Source: PLoS One. 2021 May 26;16(5):e0252124. doi: 10.1371/journal.pone.0252124 (PMC8153459; doi:10.1371/journal.pone.0252124)
Supplement: S2 Table — (PDF) [file pone.0252124.s002.pdf]

Supplementary Table 2. Medication regimens among treatment groups

Treatment groups	Regimens	Drug component
V+T-based	**VT**D	bortezomib, thalidomide, dexamethasone
	**VT**MD	bortezomib, thalidomide, melphalan, dexamethasone
	**VT**CD	bortezomib, thalidomide, cyclophosphamide, dexamethasone
	**VT**C	bortezomib, thalidomide, cyclophosphamide
	**VMT**	bortezomib, melphalan, thalidomide
	**VT**	bortezomib, thalidomide
V-based	**V**CD	bortezomib, cyclophosphamide, dexamethasone
	**V**C	bortezomib, cyclophosphamide
	**P**AD	bortezomib, doxorubicin, dexamethasone
	**V**D	bortezomib, dexamethasone
	**V**MD	bortezomib, melphalan, dexamethasone
	**V**M	bortezomib, melphalan
	**V**A	bortezomib, doxorubicin
	**V**	bortezomib
T -based	**T**D	thalidomide, dexamethasone
	MD**T**	melphalan, dexamethasone, thalidomide
	**T**CD	thalidomide, cyclophosphamide, dexamethasone
	M**T**	melphalan, thalidomide
	**T**	thalidomide
Non-V/T-based	vAD	vincristine, doxorubicin, dexamethasone
	CD	cyclophosphamide, dexamethasone
	MD	melphalan, dexamethasone
	D	dexamethasone
	C-vAD	cyclophosphamide, vincristine, doxorubicin, dexamethasone
	C	cyclophosphamide
	M	melphalan
	DCEp	dexamethasone, cyclophosphamide, etoposide, cisplatin
