# Supplementary material for: Survival, health care resource utilization and expenditures of first-line treatments for multiple myeloma patients ineligible for transplant in Taiwan
Source: PLoS One. 2021 May 26;16(5):e0252124. doi: 10.1371/journal.pone.0252124 (PMC8153459; doi:10.1371/journal.pone.0252124)
Supplement: S3 Table — (PDF) [file pone.0252124.s003.pdf]

Supplementary Table 3. Selected comorbidities and associated International Classification of Disease, 9th and 10th editions, Clinical Modification (ICD-9-CM and ICD-10-CM) codes

Comorbidities	ICD-9-CM code	ICD-10-CM code	Description
Cardiovascular disease (CVD)	410.x, 412.x, 413.x, 414.x, 429.2	I20.x, I21.x, I22.x, I25.x	Coronary artery disease
	398.91, 402.01, 402.11, 402.91, 404.01, 404.03, 404.11, 404.13, 404.91, 404.93, 425.4-425.9, 428.x	I09.9, I11.0, I13.0, I13.2, I42.0, I42.5–I42.9, I43.x, I50.x, P29.0	Congestive heart failure
	426.0, 426.13, 426.7, 426.9, 426.10, 426.12, 427.0-427.4, 427.6-427.9, 785.0, 996.01, 996.04, V45.0, V53.3	I44.1–I44.3, I45.6, I45.9, I47.x–I49.x, R00.0, R00.1, R00.8, T82.1, Z45.0, Z95.0	Arrhythmia
Diabetes mellitus (DM)	250.x0<br>250.x2	E08.x, E09.x, E11.x, E13.x	Type 2 DM (with or without mention of complications)
Peripheral neuropathy (including neuritis)	053.13, 072.72	B02.23, B26.84	Infection-related polyneuropathy (postherpetic, mumps)
	337.0, 337.1, 356.4, 356.8, 356.9	G60.3, G60.8, G60.9, G90.0, G99.0	Idiopathic neuropathy
	356.0, 356.2	G60.0, G60.2	Hereditary neuropathy
	357.x	E08.40-E08.42, E09.40-E09.42, E10.40- E10.42, E11.40-E11.42, E13.40-E13.42, G13.x, G61.x, G62.x, G63, G65.x	Disease and drug-related neuropathy
	377.3x, 377.4x	H46.x, H47.0	Optic neuropathy/neuritis
	354.x, 355.x	G56.x, G57.x, G58.x, G59.x	Mononeuritis
	729.2	M54.10, M79.2	Neuralgia, neuritis and radiculitis, unspecified
Ischemic stroke	433.x,<br>434.x	I63.x, I65.x, I66.x	Vertebral, carotid, pre-cerebral arteries<br>Cerebral arteries
Venous thromboembolism disease	415.1x	I26.x, I27.82	Pulmonary embolism
	451.x, 453.x	I80.x, I82.x	Deep vein thrombosis
	452.x	I81.x	Portal vein embolism
Osteoporosis	733.0	M81.x	Osteoporosis

Chronic obstructive pulmonary disease	491.x,<br>492.x,<br>496.x	J41.x, J42.x, J43.x, J44.x	Chronic bronchitis<br>Emphysema<br>Chronic airways obstruction
Arthritis	714.x, except 714.81	M05.x, M06.x, M08.x, M12.0	Rheumatoid arthritis<br>(714.81: Rheumatoid lung)
	715.x	M15.x-M19.x	Osteoarthritis
