# Supplementary material for: Survival, health care resource utilization and expenditures of first-line treatments for multiple myeloma patients ineligible for transplant in Taiwan
Source: PLoS One. 2021 May 26;16(5):e0252124. doi: 10.1371/journal.pone.0252124 (PMC8153459; doi:10.1371/journal.pone.0252124)
Supplement: S4 Table — (PDF) [file pone.0252124.s004.pdf]

Supplementary Table 4. Charlson Comorbidity Index (CCI) and related diagnosis codes

Comorbidity	ICD-9-CM code	ICD-10-CM code
Myocardial infarction	410.x, 412.x	I21.x, I22.x, I25.2
Chronic heart failure	428.x	I09.9, I11.0, I13.0, I13.2, I25.5, I42.0, I42.5-I42.9, I43.x, I50.x, P29.0
Peripheral vascular disease	443.9, 441.x, 785.4, V43.4	I70.x, I71.x, I73.1, I73.8, I73.9, I77.1, I79.0, I79.2, K55.1, K55.8, K55.9, Z95.8, Z95.9
Cerebrovascular disease	430.x-438.x	G45.x, G46.x, H34.0, I60.x–I69.x
Dementia	290.x	F00.x–F03.x, F05.1, G30.x, G31.1
Chronic pulmonary disease	490.x–505.x, 506.4	I27.8, I27.9, J40.x–J47.x, J60.x–J67.x, J68.4, J70.1, J70.3
Rheumatic disease	710.0, 710.1, 710.4, 714.0–714.2, 714.81, 725.x	M05.x, M06.x, M31.5, M32.x–M34.x, M35.1, M35.3, M36.0
Peptic ulcer disease	531.x–534.x	K25.x–K28.x
Mild liver disease	571.2, 571.4–571.6	B18.x, K70.0–K70.3, K70.9, K71.3–K71.5, K71.7, K73.x, K74.x, K76.0, K76.2–K76.4, K76.8, K76.9, Z94.4
DM without complications	250.0–250.3, 250.7	E10.0, E10.1, E10.6, E10.8, E10.9, E11.0, E11.1, E11.6, E11.8, E11.9, E12.0, E12.1, E12.6, E12.8, E12.9, E13.0, E13.1, E13.6, E13.8, E13.9, E14.0, E14.1, E14.6, E14.8, E14.9
DM with complications	250.4–250.6	E10.2–E10.5, E10.7, E11.2–E11.5, E11.7, E12.2–E12.5, E12.7, E13.2–E13.5, E13.7, E14.2–E14.5, E14.7
Hemiplegia /paraplegia	344.1, 342.x	G04.1, G11.4, G80.1, G80.2, G81.x, G82.x, G83.0–G83.4, G83.9
Renal disease	582.x, 583–583.7, 585.x, 586.x, 588.x	I12.0, I13.1, N03.2–N03.7, N05.2–N05.7, N18.x, N19.x, N25.0, Z49.0–Z49.2, Z94.0, Z99.2
Cancer (any malignancy, except MM and MM-related disease)	140.x–172.x, 174.x.–195.8, 200.x–208.x	C00.x–C26.x, C30.x–C34.x, C37.x–C41.x, C43.x, C45.x–C58.x, C60.x–C76.x, C81.x–C85.x, C88.x, C90.x–C97.x
	MM:[a] 203.0, 203.1	C90.0, C90.1
	MM-related disease:[b] 170, 202, 203.8	C40.x, C41.x, C82.x, C83.x, C84.0, C84.1, C84.4, C84.9, C84.A, C84.Z, C85.x, C86.x, C88.4, C91.4, C96.0, C96.2, C96.4, C96.9, C96.A, C96.Z, C90.2, C90.3, C88.1, C88.2, C88.3, C88.8
Moderate or severe liver disease	456.0–456.21, 572.2–572.8	I85.0, I85.9, I86.4, I98.2, K70.4, K71.1, K72.1, K72.9, K76.5, K76.6, K76.7
Metastatic solid tumor	196.x–199.1	C77.x–C80.x
AIDS/HIV	042.x–044.x	B20.x–B22.x, B24.x

[a] These codes were all excluded for CCI calculation.

[b] These codes were excluded for CCI calculation only if they were not found after MM diagnosis.
