# Supplementary material for: Survival, health care resource utilization and expenditures of first-line treatments for multiple myeloma patients ineligible for transplant in Taiwan
Source: PLoS One. 2021 May 26;16(5):e0252124. doi: 10.1371/journal.pone.0252124 (PMC8153459; doi:10.1371/journal.pone.0252124)
Supplement: S5 Table — (PDF) [file pone.0252124.s005.pdf]

Supplementary Table 5. Common regimens in each first-line treatment group

	Number	% of total population	% of each group
All patients	1,511	100	-
V+T-based treatment group	747	49.4	100.0
VTD	471	31.2	63.1
VTMD	93	6.2	12.4
VTCD	46	3.0	6.2
VMT	33	2.2	4.4
VT	32	2.1	4.3
VTD followed by VTCD	16	1.1	2.1
Others	56	3.7	7.5
V-based treatment group	303	20.1	100.0
VD	118	7.8	38.9
VMD	82	5.4	27.1
VM	54	3.6	17.8
VCD	28	1.9	9.2
Others	21	1.4	6.9
T-based treatment group	321	21.2	100.0
TD	106	7.0	33.0
MT	100	6.6	31.2
MDT	49	3.2	15.3
T	42	2.8	13.1
Others	24	1.6	7.5
Non-V/T-based treatment group	140	9.3	100.0
D	85	5.6	60.7
M	20	1.3	14.3
Others	35	2.3	25.0

C=cyclophosphamide; D=dexamethasone; M=melphalan; T=thalidomide; V=bortezomib.
