# Supplementary material for: Survival, health care resource utilization and expenditures of first-line treatments for multiple myeloma patients ineligible for transplant in Taiwan
Source: PLoS One. 2021 May 26;16(5):e0252124. doi: 10.1371/journal.pone.0252124 (PMC8153459; doi:10.1371/journal.pone.0252124)
Supplement: S6 Table — (PDF) [file pone.0252124.s006.pdf]

Supplementary Table 6. Full Cox proportional hazards model analysis for overall survival (OS)

Variables	Hazard ratio (95% CI)	*p*-value
**Age (years) when receiving the 1LOT**		
20-49	0.89 (0.59-1.35)	0.592
50-64	Reference	
65-79	**0.83 (0.70-0.99)**	**0.040**
≥80	**1.29 (1.06-1.57)**	**0.012**
**Female (vs. male)**	**0.81 (0.71-0.94)**	**0.004**
**Initial Durie-Salmon staging**		
Stage III	Reference	
Stage II	0.82 (0.64-1.06)	0.123
Stage I	**0.50 (0.35-0.70)**	**<.001**
Missing	0.87 (0.75-1.00)	0.054
**Accessible to AuHSCT**	0.88 (0.76-1.02)	0.098
**Charlson Comorbidity Index**		
0	Reference	
1	1.19 (0.95-1.49)	0.138
2	1.20 (0.96-1.50)	0.115
≥3	**1.56 (1.27-1.93)**	**<.001**
**Comorbidities**		
Cardiovascular disease	**1.23 (1.07-1.42)**	**0.004**
Type 2 diabetes mellitus	0.95 (0.81-1.11)	0.513
Peripheral neuropathy	1.01 (0.80-1.26)	0.960
Ischemic stroke	1.22 (0.90-1.65)	0.199
Venous thromboembolism	0.87 (0.39-1.94)	0.726
Osteoporosis	0.96 (0.80-1.15)	0.673
Chronic obstructive pulmonary disease	1.14 (0.93-1.40)	0.213
Arthritis	0.99 (0.85-1.16)	0.942
**1LOT regimen**		
V+T-based	Reference	
V-based	0.96 (0.80-1.16)	0.693
T-based	**1.42 (1.20-1.68)**	**<.001**
Non-V/T-based	**1.55 (1.24-1.95)**	**<.001**

1LOT=first lines of therapy; AuHSCT=autologous hematopoietic stem cell transplantation.
