# Supplementary material for: Survival, health care resource utilization and expenditures of first-line treatments for multiple myeloma patients ineligible for transplant in Taiwan
Source: PLoS One. 2021 May 26;16(5):e0252124. doi: 10.1371/journal.pone.0252124 (PMC8153459; doi:10.1371/journal.pone.0252124)
Supplement: S7 Table — (PDF) [file pone.0252124.s007.pdf]

**Supplementary Table 7. Full Cox proportional hazards model analysis for event-free survival (EFS)**

| Variables | Hazard ratio (95% CI) | *p*-value |
|---|---|---|
| **Age (years) when receiving the 1LOT** | | |
| 20-49 | **0.66 (0.46-0.95)** | **0.026** |
| 50-64 | Reference | |
| 65-79 | 0.89 (0.77-1.02) | 0.093 |
| ≥80 | 1.01 (0.85-1.20) | 0.922 |
| **Female (vs. male)** | **0.86 (0.77-0.97)** | **0.011** |
| **Initial Durie-Salmon staging** | | |
| Stage III | Reference | |
| Stage II | 0.83 (0.68-1.02) | 0.079 |
| Stage I | **0.58 (0.45-0.75)** | **<.001** |
| Missing | 0.89 (0.79-1.01) | 0.065 |
| **Accessible to AuHSCT** | 0.90 (0.80-1.02) | 0.096 |
| **Charlson Comorbidity Index** | | |
| 0 | Reference | |
| 1 | **1.24 (1.03-1.48)** | **0.022** |
| 2 | 1.10 (0.92-1.31) | 0.316 |
| ≥3 | **1.33 (1.12-1.58)** | **0.001** |
| **Comorbidities** | | |
| Cardiovascular disease | 1.08 (0.96-1.22) | 0.203 |
| Type 2 diabetes mellitus | 0.97 (0.85-1.11) | 0.688 |
| Peripheral neuropathy | 1.05 (0.87-1.27) | 0.601 |
| Ischemic stroke | 0.93 (0.71-1.23) | 0.625 |
| Venous thromboembolism | 1.55 (0.87-2.77) | 0.138 |
| Osteoporosis | 0.97 (0.84-1.13) | 0.701 |
| Chronic obstructive pulmonary disease | 1.01 (0.84-1.22) | 0.910 |
| Arthritis | 1.05 (0.92-1.19) | 0.500 |
| **1LOT regimen** | | |
| V+T-based | Reference | |
| V-based | **1.32 (1.14-1.53)** | **<.001** |
| T-based | **1.63 (1.41-1.88)** | **<.001** |
| Non-V/T-based | **1.62 (1.32-1.98)** | **<.001** |

1LOT=first lines of therapy; AuHSCT=autologous hematopoietic stem cell transplantation.
