# Supplementary material for: Survival, health care resource utilization and expenditures of first-line treatments for multiple myeloma patients ineligible for transplant in Taiwan
Source: PLoS One. 2021 May 26;16(5):e0252124. doi: 10.1371/journal.pone.0252124 (PMC8153459; doi:10.1371/journal.pone.0252124)
Supplement: S8 Table — (PDF) [file pone.0252124.s008.pdf]

Supplementary Table 8. One-year actual cost, predicted cost, and cost ratio after commencement of first-line treatment

Type of costs	Regimen group	Actual cost (PPPM)		Predicted cost (PPPM)	
		Mean	SD	Mean	Cost ratio (95% CI)
**Total costs**	V+T-based	5,553	4,241	5,367	Reference
	V-based	5,963	4,738	5,963	1.11 (1.00-1.24)
	T-based	4,587	6,140	4,587	**0.85 (0.77-0.95)**
	Non-V/T-based	4,339	5,442	4,083	**0.76 (0.65-0.88)**
**OPD costs**	V+T-based	3,266	3,171	3,365	Reference
	V-based	3,517	3,471	3,634	1.08 (0.95-1.22)
	T-based	1,447	2,790	1,390	**0.41 (0.33-0.51)**
	Non-V/T-based	1,239	3,416	1,021	**0.30 (0.19-0.45)**
**ER costs**	V+T-based	73	131	74	Reference
	V-based	103	484	91	1.22 (0.79-1.76)
	T-based	94	241	103	1.39 (0.97-1.88)
	Non-V/T-based	83	252	90	1.21 (0.73-1.82)
**IPD (hospitalization) costs – all**	V+T-based	2,213	3,674	2,269	Reference
	V-based	2,342	4,291	2,234	0.99 (0.77-1.23)
	T-based	3,046	5,979	3,005	**1.33 (1.05-1.68)**
	Non-V/T-based	3,017	4,287	2,925	1.29 (0.97-1.70)
**IPD (hospitalization) costs – drug-related**	V+T-based	886	1,516	925	Reference
	V-based	837	1,454	840	0.91 (0.71-1.15)
	T-based	771	3,769	676	**0.73 (0.53-0.99)**
	Non-V/T-based	563	1,122	508	**0.55 (0.39-0.75)**
**IPD (hospitalization) costs – non-drug-related**	V+T-based	1,327	2,360	1,360	Reference
	V-based	1,506	3,102	1,402	1.04 (0.78-1.32)
	T-based	2,275	3,704	2,290	**1.69 (1.32-2.11)**
	Non-V/T-based	2,454	3,470	2,423	**1.79 (1.33-2.36)**

The costs are presented in US dollars.

CI=confidence interval; ER=emergency room; IPD=inpatient department; OPD=outpatient department; PPPM=per patient per month; SD=standard deviation.
